# The whole day matters, but so do living conditions: reframing the 24-hour movement through an equity lens

**DOI:** 10.1186/s12966-026-01992-z

**Published:** 2026-10-13

**Authors:** Rodrigo Reis

**Affiliations:** 1 Bursky School of Public Health, Washington University in St. Louis, 1 Brookings Drive, St. Louis, MO 63130 USA; 2 People, Health and Place Unit, Prevention Research Center, Washington University in St. Louis, One Brookings Drive, MSC 1196-257-220, St. Louis, MO 63130 USA

**Keywords:** Physical activity, Sedentary behavior, Sleep, Health equity, Social determinants of health, Commercial determinants of health, Low- and middle-income countries, Living conditions

## Abstract

The 24-hour movement approach has strengthened physical activity science by integrating physical activity, sedentary behavior, and sleep within the day’s finite time. This Comment argues that its major contribution is also its main limitation. By treating the day as a composition of movement states, the approach extends the energy-expenditure definition of physical activity into a more complete, yet still behavior-centered, account of daily life. Physical activity should also be understood as a population condition shaped by governments, institutions, and markets through access to opportunity. This shift matters because individuals do not simply allocate movement, sitting, and sleep; labor, transport, housing, safety, gender, income, climate, commercial forces, and public infrastructure structure these activities. Moreover, in low-income settings and low- and middle-income countries, necessity often shapes activity, while choice-constrained conditions frequently limit leisure-time or volitional activity. A more equitable 24-hour framework should therefore move from movement behaviors alone to 24-hour living conditions, asking not only how time is spent but also how opportunity is distributed.

## Main text

Physical activity research has been shaped over the past four decades by a definition that is both biologically precise and epidemiologically useful, and by the tools used to operationalize it. Self-report instruments, accelerometers, wearable devices, and, more recently, measures of sedentary behavior and sleep have each influenced what the field sees and values. Caspersen and colleagues defined physical activity as bodily movement produced by skeletal muscles that results in energy expenditure [1]. This definition helped distinguish physical activity from exercise and fitness and made the field measurable. However, it also narrowed the understanding of the phenomenon of interest. Once physical activity is defined primarily by bodily movement and energy expenditure, the social conditions under which people move can become secondary to the amount, intensity, and duration of movement.

Hallal and colleagues recently offered an important correction to this narrow understanding. Drawing on Rose’s distinction between sick individuals and sick populations, they argue that physical activity should be viewed not only as an individual attribute but also as a property of populations [2]. Their proposed complement to Caspersen’s definition is deliberately political: governments and institutions are responsible for ensuring access to pleasant, safe, healthy, equitable, purposeful, and, when possible, enjoyable opportunities for activity in everyday life [2]. This addition matters because it shifts responsibility from individuals’ motivation alone to opportunity, from prescription alone to conditions, and from individuals’ alignment with guidelines to population-level accountability.

The 24-hour movement approach should be understood in this historical context. This emerging movement has made a genuine contribution to the field by integrating physical activity, sedentary behavior, and sleep into a single daily cycle and correcting the artificial separation of behaviors that compete for the same finite time [3]. Compositional approaches have further strengthened this insight by showing that time spent in one behavior is time displaced relative to another [4]. The field therefore rightly assumes that the whole day matters. However, contemporary 24-hour movement measurement often translates that insight into mutually exclusive categories. This differs from broader time-use traditions, which can capture concurrent activities, specific tasks, location, social context, and co-presence. Watching television while stretching, supervising a child while cooking, waiting for transport while using a phone, or walking for work while exposed to traffic risk are not just time allocations; they are lived activities embedded in place, relationship, and obligation.

Yet the whole day is not lived in a vacuum, disconnected from daily living conditions. Notably, a 24-hour composition describes how time is distributed, not why or under what circumstances. Two people may share similar daily patterns of sleep, sedentary time, light activity, and moderate-to-vigorous physical activity, yet live profoundly different lives. For one, active transport may be a safe, chosen routine; for another, it may be the only affordable option on unsafe and unpleasant routes. For one, sedentary time may be a leisure practice of choice; for another, it may reflect unemployment, disability, unavailability of public spaces, digital labor, or precarious work. For one, short sleep may reflect preference or a life transition; for another, it may stem from overcrowded housing, night shifts, caregiving, excessive heat, noise, or fear. Without these distinctions, the 24-hour movement approach risks treating structurally produced patterns as behavioral choices.

This is not merely a measurement problem but a conceptual one with practical consequences for population health. When social determinants appear in 24-hour movement research, they are often treated as correlates, covariates, or subgroup descriptors. Although this is useful, it is insufficient. A recent scoping review of socioeconomic indicators and their alignment with 24-hour movement guidelines among children and adolescents found limited literature, with indicators most often limited to parental education and household income, and inconclusive findings [5]. More concerning is not that inequalities are seldom measured, but that they are rarely placed at the center of the causal story.

An equity perspective raises questions that go beyond merely measuring socio-demographic characteristics. It asks how socioeconomic, political, and cultural contexts shape daily living conditions and how those conditions, in turn, shape individual behaviors [6]. When applied to 24-hour movement, this perspective asks how schools, transportation systems, housing, labor markets, public safety, caregiving arrangements, gender norms, racism, disability, migration status, climate exposure, and commercial determinants organize the possibilities for moving, sitting, resting, and sleeping. Commercial determinants are particularly relevant because corporate and market practices can make motorized travel, screen-based entertainment, precarious platform work, and privatized sports and recreation more accessible than safe active travel and free public space [7, 8]. Therefore, although the unit of analysis remains the day, the focus shifts to the lived conditions that make up the day.

This critique does not reject 24-hour movement or its contribution to the science of physical activity. It argues for fulfilling its fundamental assumptions. If movement behaviors are interdependent, the conditions that produce them are as well. Sleep is connected to housing and labor. Sedentary behavior is connected to fundamental living conditions and systems, including work, schooling, transportation, safety, digital environments, disability, and commercial design. Physical activity is connected to land use, gender relations, income, policing, climate, and the design of streets and public space. A framework that integrates behaviors but fragments, and at times does not consider the conditions that produce them, remains only partially integrated.

This reframing is especially important in low-income settings and low- and middle-income countries (LMICs). In many contexts, movement is not an optional lifestyle behavior. It is embedded in informal work, domestic labor, active transport, and survival strategies. Salvo and colleagues’ necessity-versus-choice framework makes this clear: active transport and occupational activity may be driven by economic necessity rather than by free, health-enhancing choice [9]. Moreover, choice-based physical activity also warrants closer scrutiny. Describing leisure-time activity as a choice can obscure constrained conditions: people may value and even desire certain activity yet lack money, time, safety, childcare, transport, public space, or social permission to participate. In other words, they would if they could, but they cannot. New global evidence on domain-specific physical activity inequalities further underscores why domain and choice matter: active leisure, active transport, and active labor have distinct social meanings and equity implications [10].

The implications of this reframing for intervention and policy translation are significant. The 24-hour movement paradigm should not merely repackage individual-level behavior-change interventions; it should inform people about ways to rebalance their day that may be meaningful when they have control over time, safety, and resources. However, this approach is less meaningful when poverty, transportation disadvantage, violence, shift work, care burdens, or environmental hazards govern time. In such settings, a 24-hour movement intervention cannot simply ask individuals to sit less, sleep more, or accumulate more moderate-to-vigorous activity. It must ask what would make these changes possible. The intervention target may be a sidewalk, bus route, protected crossing, safe park, school timetable, workplace regulation, housing policy, social protection, protection from violence, or climate-resilient public space.

Operationalizing a 24-hour living-conditions approach does not require waiting for a perfect framework, as early actions are available. Studies can report whether movement profiles are compatible with health and well-being, not only whether guideline thresholds are met. Interventions can pair universal messages with proportionate supports, an approach already visible in equity-oriented health promotion practice, including uses of proportionate universalism, in which population-wide strategies are intensified for disadvantaged communities. A school-based 24-hour intervention, for example, could combine active breaks with safe routes to school, after-hours access to playgrounds, and support for families facing housing stress. An active travel intervention could combine cycling promotion with traffic calming, lighting, affordable public transportation, and protection from harassment.

Surveillance and guidelines also need this shift and can include brief items on domains such as autonomy, safety, location, care responsibilities, shift work, and access to free or low-cost places for activity. The next generation of 24-hour movement research should not only estimate time spent in movement states but also characterize domains of autonomy, safety, dignity, and structural opportunity. It should distinguish among chosen, constrained, coerced, and necessity-driven movement. It should ask whether physical activity is health-enhancing or exhausting, pleasurable or stressful, safe or hazardous, and socially supported or stigmatized. It should treat sleep not only as duration but as a condition shaped by housing, work, care, and security. It should treat sedentary time not only as a risk but as a heterogeneous behavior with different meanings across class, gender, age, disability, and work.

## Conclusions

The 24-hour movement approach has usefully broadened the scope of physical activity science, while also adding complexity to measurement and public health messaging about physical activity, sedentary behavior, sleep, and their health, social, and developmental benefits. The next step is to broaden the social and political scope. If the whole day matters, the conditions that organize it must matter as well. A field that counts movement without explaining opportunity risks mistaking inequality for lifestyle. Shifting from 24-hour movement behaviors to 24-hour living conditions would make the approach more relevant to equity, more useful for real-world intervention, and more accountable to populations for whom physical activity is not only a matter of behavior but also of rights, safety, and possibility.

## Data Availability

No datasets were generated or analysed during the current study.

## Abbreviations

LMICs: Low- and middle-income countries
